# Pulmonary arterial hypertension in Latin America. The age and comorbidity paradox

**DOI:** 10.1016/j.ijcchd.2025.100573

**Published:** 2025-01-31

**Authors:** Tomas Pulido, Sofia de la Cruz-Perez, Daniela Valencia, Rafael Conde, Adrian Lescano, Nayeli Zayas

**Affiliations:** a From the Clinical Research Department, Instituto Nacional de Cardiologia Ignacio Chavez, Mexico City, Mexico; b From the Systematic Support Office for Higher Research (OASIS), Instituto Nacional de Cardiologia Ignacio Chavez, Mexico City, Mexico; c From the Pulmonary Vascular Diseases Program, Fundación Neumológica Colombiana and Fundación Cardioinfantil de Bogota, Colombia; d From the Cardiology Department Fundacion Favaloro and Centro Gallego, Buenos Aires, Argentina; e From the Cardiopulmonary Department, Instituto Nacional de Cardiologia Ignacio Chavez, Mexico City, Mexico

## Abstract

Pulmonary arterial hypertension (PAH) has been classically described as a disease in young adults, predominantly females with no comorbidities. However, in recent registries, the epidemiology has changed to older patients with comorbidities such as obesity, diabetes, systemic hypertension, and coronary heart disease. Nevertheless, there is not enough inclusion of these patients in clinical trials. In contrast, in Latin America, registries have shown that PAH patients are younger and have fewer comorbidities, which raises the question of whether Latin American patients present a different phenotype or if we are lagging behind developed countries and whether we will experience a change in epidemiology in the next couple of years. We analyzed these trends in this review.

## 1 Introduction

Pulmonary Arterial Hypertension (PAH) is a disease of the pulmonary vasculature characterized by endothelial dysfunction and an imbalance of endothelium-derived vasoconstrictors and vasodilators, leading to vascular proliferation [1]. This condition eventually leads to an increase in the mean pulmonary arterial pressure (mPAP) > 20 mmHg at rest; the primary characteristics of the pathological parameters include plexiform lesions, which appear in histopathological investigations; increased pulmonary vascular resistance (PVR >2 Wood units); and pulmonary capillary wedge pressure (PCWP) or left ventricular end-diastolic pressure (LVDP) ≤ 15 mmHg [2]. Symptomatic PAH may precede signs by several years because of the relatively irreversible nature of these parameters, emphasizing the need for early recognition and intervention.

Typically, PAH, specifically idiopathic pulmonary arterial hypertension (iPAH), is a disease that predominantly affects young females without comorbidities. Recent multicenter registries have shown changes in demography and clinical characteristics. As patients have a better survival rate, they are becoming older, and comorbidities have appeared. However, in Latin America (LATAM), registries still include younger patients without too many comorbidities. This review analyzes the differences in demographics and patient responses in LATAM.

## 2 The changing demography of PAH

Patient registries are essential for capturing the clinical landscape of PAH and informing patient care. Hoeper and Gibbs emphasized the critical role of PAH registries in collecting demographic, clinical, and hemodynamic data, which has been instrumental in understanding the epidemiology and survival rates of patients with PAH. They noted a shift in demographics, indicating that PAH is increasingly diagnosed in older patients, which has implications for treatment and management strategies [3].

In the 1981 US registry of 187 patients, the mean age was 36 ± 15 years, and the female-to-male ratio was 1.7:1 [4]. The median survival time was 2.8 years from diagnosis, and death was associated with right heart failure [5]. Despite this, the demographics have changed; patients are older and have more comorbidities [[6], [7], [8], [9], [10]]. (Table 1). For example, in the Registry to Evaluate Early And Long-term PAH disease management (REVEAL) registry [6], the average age of the first 2967 consecutively enrolled patients was 53 ± 14.5 years, and the female-to-male ratio was 3:1. Among those with PCWP ≤15 mmHg, 33.3 % were obese, 40.2 % had systemic hypertension, 12 % had diabetes, and 21 % had obstructive sleep apnea (OSA). In the Comparative, Prospective Registry of Newly Initiated Therapies for Pulmonary Hypertension (COMPERA) registry [7], out of 2079 patients, 1120 had a median age of 72 (52–78) years and comorbidities such as systemic hypertension, coronary heart disease, diabetes, and body mass index (BMI) > 30 kg/m^2^. Among those with pulmonary hypertension in the PoLish population (BNP-PL) [8], of the 444 patients with PAH, 50 % had systemic hypertension, and 37 % were obese. Importantly, the number of comorbidities strongly influences patient improvement with targeted therapy for PAH and survival. In contrast, since 1995, when the first targeted drug for PAH was approved (epoprostenol, Epo) [11], the demographics of subjects enrolled in clinical trials have not changed significantly with respect to age and sex, from an average age of 40 ± 2 years in 1995 and 70 % females without comorbidities to 47.9 ± 14.8 years and 79.3 % female subjects in the most recent trial of sotatercept (STELLAR) [12]. However, in the STELLAR study, the average BMI was 26.4 ± 5.9 kg/m^2^, and 22.9 % of patients had a BMI of ≥30 kg/m^2^ [12]. Additionally, in a recent analysis of secular and regional trends among PAH clinical trials, it was concluded that even though there were no significant changes in sex, patients enrolled in more recent PAH therapy trials were older and more obese, mirroring the changing epidemiology of observational cohorts [13]. The comorbidity issue is essential since conflicting opinions have been published, and multiparametric risk stratification tools may not reflect these patients' actual risk [14].

**Table 1:** PAH Registries along the years.

Registry (ref.)	Year	# Subjects	Age	Country
PPH NIH (4)	1987	187	36 ± 15	USA
INCICH (17)	1994	61	22.6 ± 11	Mexico (one center)
IPPHS (36)	1996	95	44.7 ± 12.3	Europe
French PAH (29)	2006	264	52 ± 15	France
PHC PAH (38)^a^	2010	576	48 ± 14	USA (one center)
REVEAL PAH (6)	2010	1166	53.±14.5	USA
UK IPAH (8)^a^	2012	482	50.1 ± 17.1	UK
ASPIRE (42)	2012	598	54 ± 18	UK (one center)
REHAP (43)	2012	314	46 ± 18	Spain
COMPERA IPAH (7)	2012	587	71 ± 16	Europe, UK
Registry of Incident Brazilian Cases (21)	2015	51	39.8 ± 14.8	Brazil
SPAHR (39)	2016	227	69 ± 21	Sweden
HOPE (40)	2018	77	55.1 ± 24.1	Greece
Latvian PAH (44)	2018	130	65 (47–71)	Latvia (one center)
BNP-PL (8)	2020	444	54.6 ± 18.2	Poland
iPHNET registry^a^ (45)	2021	181	53 ± 16	Italy
RECOPILAR (22)	2021	399	47 ± 18	Argentina
HAPred.co (16)	2022	682	50.6 ± 18	Colombia
REMEHIP (18)	2024	619	43 ± 15	Mexico

a Any pulmonary artery hypertenson aetiology.

## 3 Latin American demographics

Information about the demographics of PAH patients in Latin America is scarce. This region has been largely overlooked in disease reviews despite reported differences in patient profiles compared with other areas [13,15]. Many of these data are from single centers or minor registries. The first published registry in Latin America (1994) was that of Sandoval et al. [16], which included 61 children and adults from a referral center in Mexico City. The average age was 22.6 ± 11 years, and 75.4 % were females. Differences in survival among US and Mexican patients were noted: the median survival times were 2.8 and 4.08 years, respectively, suggesting an ethnic difference between the US and Mexico [5,16]. On the other hand, in the recently published Mexican Registry of Pulmonary Hypertension (REMEHIP), the median age was 43 ± 17 years, and 86 % of the patients were female [17] (Table 2). Additionally, we have recently shown in a survey of 958 PAH patients whose average number of comorbidities was 2.4, where 239 (25 %) patients had systemic hypertension and 153 were obese [18], contrary to the COMPERA registry, in which out of 1120 patients, only 208 (18.5 %) had no comorbidities and 66.6 % had systemic hypertension [[19], [20]].

**Table 2:** Evolution of demographic characteristics in Mexico 1994 vs 2024.

Characteristic	Sandoval J, (ref. 17) N = 61	REMEHIP (ref. 18) N = 619	p-value
Age, years (mean ± SD)	23 ± 11	43 ± 15	<0.001^a^

Sex (% Female)	(75.4)	(81.9)	0.065^b^
NYHA/WHO FC n (%)			<0.001^b^
I	16 (26)	64 (10.8)	
II	21 (34.4)	330 (55.7)	
III	20 (33)	186 (31.4)	
IV	4 (6.6)	12 (2)	
RAP, mmHg (mean ± SD)	7.6 ± 5	8.5 ± 5	0.215^a^
mPAP, mmHg (mean ± SD)	65.5 ± 17.5	55 ± 18	<0.001^a^
PCWP, mmHg (mean ± SD)	7.5 ± 4.5	10 ± 5	<0.001^a^
CI, L.min/m^b^ (mean ± SD)	3.15 ± 1.3	3.0 ± 1.3	0.426^a^
Time to diagnosis, years	3.8	1.5	<0.001^a^

Abv. NYHA/WHO FC: New York Heart Association/World Health Organization functional class; RAP: right atrial pressure; mPAP: mean pulmonary arterial pressure; PCWP: pulmonary capillay wedge pressure; CI: cardiac index.

a Welch Two Sample *t*-test.

b Pearson's chi squared test.

By analyzing published Latin American registries, we can observe similarities in age, severity of disease, and comorbidities among these patients. Among the 1751 patients included, the average age was 47 ± 17 years, 75 % were female, and 25 % had at least one comorbidity; in contrast to the European registries, this means that in LATAM, we are evaluating patients with more “typical” PAH (Table 3, Table 4). One explanation for this could be that in most LATAM countries, there are only a limited number of referral centers; thus, once PAH is suspected, patients are sent to one of these centers; in other words, a diagnosis is made in the early stages. This can be corroborated with data from the REMEHIP registry and the Brazilian registry, where 66.6 % and 47 % of the patients were in functional class (FC) I and II, respectively [17,21], contrary to the COMPERA and the Reveal registries [6,7]. However, in Argentina, only 35 % of patients were in the early stages of the disease. (Table 3). As world demographics change, we may see older patients with more comorbidities in LATAM in the next few years [[22], [23], [24]].

**Table 3:** Comparison of European and US vs LATAM Registries.

Registry^(ref)^	France^29^ N = 264	REVEAL^6^ N = 1166	Spain^42^ N = 314	UK^43^ N = 598	COMPERA^7^ N = 587	Sweden^39^ N = 227	Greece^40^ N = 77	Latvia^44^ N = 130	Poland^8^ N = 444	Europe and US N = 3807	Brazil^21^ N = 51	Argentina^22^ N = 399	Colombia^16^ N = 682	Mexico^18^ N = 619	Latin America N = 1751	p-value
Age, years (mean ± SD)	52 ± 15	53 ± 15	46 ± 18	54 ± 18	71 ± 16	69 ± 21	55 ± 24	61 ± 18	55 ± 18	57 ± 19	40 ± 15	47 ± 18	50.6 ± 18	43 ± 15	47 ± 17	<0.001^a^
Sex: Female	164(6.1)	936 (80.3)	229(73)	419 (70)	354 (60.3)	125 (55)	48 (62.3)	95 (73)	318 (72)	2688(71)	40 (78)	314 (79)	464 (68)	507(81.9)	1315 (75)	<0.001^b^
NYHA/WHO FC III/IV	213(80.5)	594(55.3)	248(79)	466 (78)	534(91)	227 (82)	39 (50.6)	92 (72)	365 (82)	2778(73)	27 (53)	259 (65)	266 (50)^c^	198(33.4)	750 (47)	<0.001^b^
6 MW, m (mean ± SD)	328 ± 112	374 ± 129^a^	382 ± 117		293 ± 126	279 ± 216	420 ± 149	322 ± 122	383 ± 151	351 ± 142	398 ± 152	367 ± 118		378 ± 112	375 ± 117	<0.001^a^
mPAP, mmHg (mean ± SD)	56 ± 14	52 ± 13	55 ± 15	48 ± 13	44 ± 12	47 ± 14	48 ± 17	49 ± 18	45 ± 14	49 ± 14	61 ± 20	52 ± 17	50 ± 16^d^	57.3 ± 19	54 ± 18	<0.001^a^
PVR, UW (mean ± SD)	22.8 ± 10	22.9 ± 11.4^b^	12 ± 6	9.8 ± 5.6	9.6 ± 5.5	9.3 ± 5	8.8 ± 6.1	7.3 ± 4.8	8.1 ± 4.2	13.8 ± 9.9	13.5 ± 6.4	9.9 ± 6.1	9±5^d^	12.0 ± 8.1	10.6 ± 6.9	<0.001^a^

Abv. NYHA/WHO FC: New York Heart Association/World Health Organization functional class; 6 MW: 6 min walk test; mPAP: mean pulmonary arterial pressure; PVR: pulmonary vascular resistance; WU: Wood units.

^a^ N = 921; ^b^ N = 842; ^c^ N = 532; ^d^ N = 426.

a Welch Two Sample *t*-test.

b Pearson's chi squared test. p-value calculated from pooled data between Europe and US, and Latin America.

**Table 4:** Comparison of comorbidities between European and US vs LATAM registries.

Registry^(ref)^	REVEAL^6^ N = 1166	Sweden^39^ N = 227	Greece^40^ N = 77	Poland^8^ N = 444	Europe and US N = 1862	Brazil^19^ N = 306	Argentina^19^ N = 200	Colombia^19^ N = 202	Mexico^19^ N = 250	Latin America N = 958	p-value^1^
Systemic hypertension	466 (42)^a^	110 (48)	30 (39)	222 (50)	828 (44)	92 (30)	30 (15)	47(23)	68(27)	237 (25)	<0.001
Obesity	365 (38.4)		26 (33.8)	130 (37)	521(31)	34 (11)	24 (12)	44 (22)	53 (21)	153 (16)	<0.001

1 Pearson's chi squared test. p-value calculated from pooled data between Europe and US, and Latin America.

a N = 1114.

**Fig. 1:**
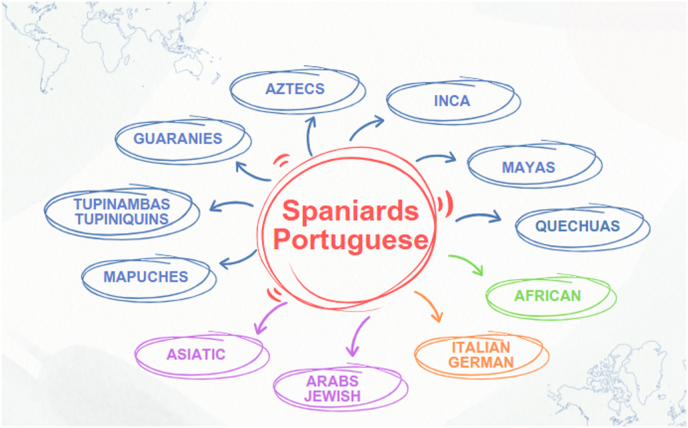
Ethnicity in Latin America is highly diverse, ranging from Latin American natives (blue), Africa, Asia, and Europe. Most European ancestry comes from Spain and Portugal.

**Fig. 2:**
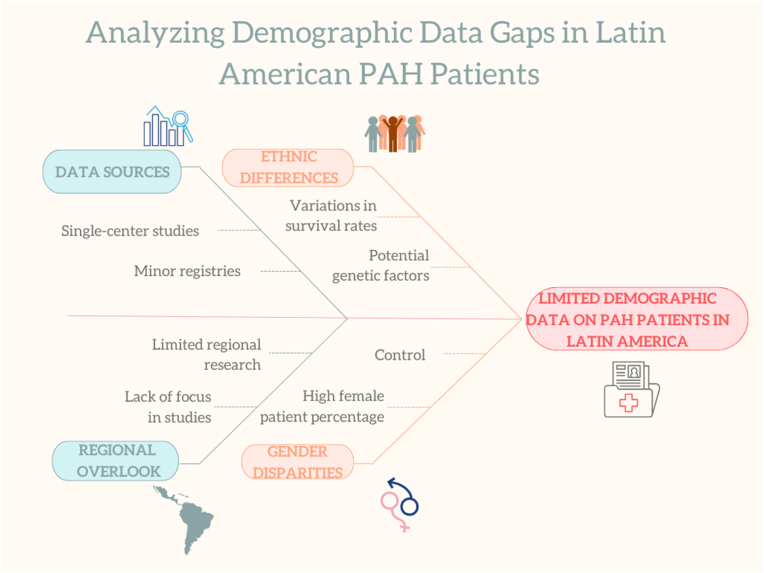
Overview of the findings, demonstrating how genetic ancestry, phenotypic variations, and gaps in genomics research are interconnected in the LATAM region. This figure underscores significant differences in health outcomes driven by systemic inflammation, highlighting the crucial requirement for enhanced representation in demographic studies to comprehend and tackle these distinct challenges effectively.

The other explanation for these differences across Europe, the USA, and LATAM could be the ethnic background of the latter region. Owing to insufficient data, Latin America continues to be severely underrepresented in genomics research [25]. Genetic ancestry or racial differences in health outcomes exist in diseases associated with systemic inflammation (e.g., COVID-19 and asthma) [26,27], and the LATAM region is highly diverse in terms of ancestry (Fig. 1). This genetic ancestry can influence the phenotypic traits of patients with PAH. Al-Naamani and colleagues [28] compared PAH patients of Hispanic origin with those of non-Hispanic ancestry and reported that Hispanics were younger and had more severe hemodynamics, similar to patients from Mexico, indicating that more genetic research is needed [17,28]. (Fig. 2).

## 4 Risk stratification in Latin American patients

The actual treatment of PAH is based on multiparametric risk assessment tools. Most of these parameters in PAH patients are the sum of several nonmodifiable risk factors, such as age, sex, and type of PAH, and modifiable factors, such as comorbidities and symptoms [29]. Tools such as REVEAL [6], REVEAL lite [30], COMPERA [7], and the one from the French registry [31] are derived from PAH registries, and except for a minority in the REVEAL registry, they do not include Hispanic patients. Fadah et al. [29] validated these three tools in Hispanic US patients and concluded that they accurately predict outcomes in treatment-naïve Hispanic patients. Recently, Diez and colleagues validated three noninvasive variables from the French registry [32] in a Latin American cohort, mainly from Argentina, and concluded that efforts should be made to achieve a low-risk profile as a treatment goal. However, as previously mentioned, PAH patients in Argentina have different demographic characteristics. Nevertheless, risk stratification tools have been effectively validated in this population [32], which shows that further studies are necessary to validate these tools in different LATAM populations [[33], [34], [35]].

## 5 Response to targeted therapy

As new drugs to target PAH have been developed, a more heterogeneous and worldwide population has been included in recent clinical trials. In an analysis of 21 clinical trials and 6599 PAH patients, Min et al. reported that only 6.6 % (n = 463) were from Latin America, with a median age of 40.9 years, which was the second youngest, followed by Asia (median age of 38.2 years), with a significant difference among European and US patients (51.8 and 51.5 years, respectively). The authors concluded that although more recent randomized clinical trials (RCTs) have improved the overall racial diversity of study participants, there is a need for high-quality, prospective, multicenter registries in Latin America and Asia to study changes in PAH epidemiology and outcomes [13]. Some publications have shown that, overall, older patients with comorbidities respond equally to younger patients without comorbidities and that US patients of different ethnicities may also have the same clinical response [36,37]. However, a recent meta-analysis of European studies revealed that patients with comorbidities, especially those with lung comorbidities, had increased mortality risk, and these patients were prescribed mainly monotherapy [38].

Information on Latin American patients included in clinical trials has also been published, revealing differences in the results obtained [39,40]. For example, in the Study with an Endothelin Receptor Antagonist in Pulmonary Arterial Hypertension to Improve Clinical Outcome (SERAPHIN) [39], where two doses of macitentan were evaluated (3 mg and 10 mg), it was concluded that the effective dose was 10 mg QD due to the lack of efficacy of the 3 mg dose in the overall population. However, the 3 mg dose was effective in LATAM patients when the populations were analyzed by geographical region. (34, suppl. material). The COMPASS-2 trial, where bosentan was added to sildenafil, was a negative trial with no difference between the active drug group and the placebo group in the overall population, except in subjects randomized from Brazil, where this combination had a beneficial effect [41]. Additionally, in the Prostacyclin (PGI2) Receptor Agonist In Pulmonary Arterial Hypertension (GRIPHON) study evaluating the safety and efficacy of selexipag [40], a more significant effect of the composite primary endpoint was achieved in patients from the LATAM region (36, suppl. material). Notably, the effectiveness described in at least two trials [39,40] is better than that reported in the Asian population, suggesting that younger patients have a better response (see above). These LATAM results could be explained only by differences in ethnicity, meaning that these patients are somewhat distinct and may respond differently to specific therapies than patients from the rest of the world do. However, more studies are needed to corroborate this theory.

In addition, there is a difference in PAH prescription across LATAM countries. The most common treatment classes prescribed are phosphodiesterase type-5 inhibitors (PDE5i) and soluble guanylate cyclase stimulators (sGCS), which are the least common, with differences in proportions between countries. For example, in Argentina, 88 % of patients receive PDE5i, 81 % receive endothelin receptor antagonist (ERA), and 3 % receive sGCS, whereas in Mexico, 81 % receive PDE5i, 25 % receive ERA, and 7 % receive sGCS. The proportion of patients receiving combination therapies also varies across countries, ranging from 21 % of patients in Mexico to 79 % of patients in Argentina. Additionally, there was a difference in the proportion of patients with prescribed combination therapy by the time of diagnosis: 23 % with no more than one year since diagnosis and 60 % with three years since diagnosis [18]. The meta-analysis mentioned above, which included only European studies, revealed that 13.9 % of patients with comorbidities and 19.1 % of patients without comorbidities received combination therapy. COMPERA revealed that the most common initial and 1 year after diagnosis was PDE5i (81 %), followed by ERA (24 % and 44 %), whereas in Greece, two-thirds of the patients were receiving therapy with PDE5i and ERA [20,38,42].

## 6 Survival

Globally, in 2021, there were 192 000 prevalent cases of PAH, and 22 000 deaths were attributed to this disease, with regional variation. Moreover, mortality has decreased worldwide [43]. For example, from 2012, when the ASPIRE registry was published, to 2018, with the HOPE registry, the 36-month survival rate increased from 63 % to 75.7 %, which can be explained by the availability of PAH treatments and timely diagnosis [42,44]. However, survival rates have remained more consistent in Latin America. In 2015, the 3-year survival rate was 87 % in Brazil, and in 2021, Argentina reported a survival rate of 82.8 % [21,45]. These regional differences might imply an uneven spread of risk factors involved in the development of PAH, including genetic predisposition.

## 7 Conclusions

PAH is a chronic condition that is traditionally observed in young females without comorbidities, with shifts in recent registries. However, LATAM patients are still relatively young and have relatively few comorbidities. Although LATAM patients are underrepresented in clinical trials, evidence suggests that they may respond differently to therapies such as macitentan and bosentan. These differences could be due to ethnic diversity, indicating the need for further research to explore regional variations in LATAM patients’ demographics, risk stratification, and treatment response.

## CRediT authorship contribution statement

**Tomas Pulido:** Writing – review & editing, Writing – original draft, Supervision, Methodology, Investigation, Conceptualization. **Sofia de la Cruz-Perez:** Writing – review & editing, Methodology, Formal analysis, Conceptualization. **Daniela Valencia:** Writing – review & editing, Visualization, Methodology, Conceptualization. **Rafael Conde:** Writing – review & editing, Methodology, Conceptualization. **Adrian Lescano:** Writing – review & editing, Methodology, Conceptualization. **Nayeli Zayas:** Writing – review & editing, Methodology, Conceptualization.

## Declaration of competing interest

The authors declare that they have no known competing financial interests or personal relationships that could have appeared to influence the work reported in this paper.
